# Structural Basis of Redox-Sensing Transcriptional Repressor Rex with Cofactor NAD^+^ and Operator DNA

**DOI:** 10.3390/ijms23031578

**Published:** 2022-01-29

**Authors:** Kang Hwa Jeong, Hyun Jin Lee, Young Woo Park, Jae Young Lee

**Affiliations:** Department of Life Science, College of Life Science and Biotechnolgy, Dongguk University-Seoul, Goyang 10326, Korea; jkh970801@dongguk.edu (K.H.J.); lhj8755@dongguk.edu (H.J.L.); pyw@b2bio.com (Y.W.P.)

**Keywords:** structural analysis, Rex, reduction–oxidation (redox) state, nicotinamide adenine dinucleotide (NAD), NAD^+^/NADH, *Thermotoga maritima*, transcriptional regulation, ternary complex

## Abstract

The transcriptional repressor Rex plays important roles in regulating the expression of respiratory genes by sensing the reduction–oxidation (redox) state according to the intracellular NAD^+^/NADH balance. Previously, we reported on crystal structures of apo, NAD^+^-bound, and NADH-bound forms of Rex from *Thermotoga maritima* to analyze the structural basis of transcriptional regulation depending on either NAD^+^ or NADH binding. In this study, the crystal structure of Rex in ternary complex with NAD^+^ and operator DNA revealed that the *N*-terminal domain of Rex, including the helix-turn-helix motif, forms extensive contacts with DNA in addition to DNA sequence specificity. Structural comparison of the Rex in apo, NAD^+^-bound, NADH-bound, and ternary complex forms provides a comprehensive picture of transcriptional regulation in the Rex. These data demonstrate that the conformational change in Rex when binding with the reduced NADH or oxidized NAD^+^ determines operator DNA binding. The movement of the *N*-terminal domains toward the operator DNA was blocked upon binding of NADH ligand molecules. The structural results provide insights into the molecular mechanism of Rex binding with operator DNA and cofactor NAD^+^/NADH, which is conserved among Rex family repressors. Structural analysis of Rex from *T. maritima* also supports the previous hypothesis about the NAD^+^/NADH-specific transcriptional regulation mechanism of Rex homologues.

## 1. Introduction

Bacteria monitor and respond to environmental oxygen levels and intracellular reduction–oxidation (redox) states by controlling the transcription of genes involved in respiratory pathways [1]. Intracellular redox reactions are involved in many biological processes, such as cellular respiration, enzymatic reactions, and energy metabolism [2,3,4]. Nicotinamide adenine dinucleotide (NAD) plays an important role as an electron carrier and a key signal for intracellular redox states with oxidized and reduced forms, NAD^+^ and NADH, respectively [5]. In addition, the re-oxidation of NADH with concomitant reduction of NAD provides the major source of ATP by reduction of oxygen in electron transport chains [6]. Therefore, NAD^+^/NADH ratio can be a critical indicator of the intracellular redox state corresponding to environmental oxygen levels.

The redox-sensing transcriptional regulator Rex is well conserved across bacterial species [7]. Rex monitors the redox state according to the intracellular NAD^+^/NADH ratio and controls the Rex regulon involved in various pathways, including cellular respiration, fermentation, oxidative stress response, and biofilm formation [2,5,8,9,10]. In particular, the relation between the metabolic pathway under Rex control and pathogenesis has been suggested in some bacteria, such as *Staphylococcus aureus*, *Enterococcus faecalis*, *Streptococcus mutans*, and *Streptococcus suis* serotype 2 (SS2) [11,12,13,14]. The Rex regulon in *Thermotoga maritima* has been identified in 12 operons that include 40 genes involved in central carbohydrate metabolism and hydrogen production [15]. Rex DNA operator sites in *T. maritima* have been experimentally demonstrated by in vitro binding assays, including whether *T. maritima* Rex (*Tma* Rex) can bind specifically to cognate Rex binding sites, and how the NAD^+^/NADH ratio affects the interaction between *Tma* Rex and its cognate operator [15].

Structural studies of Rex family proteins have shown that Rex exists as a homodimer with *N*-terminal winged helix-turn-helix motifs and *C*-terminal NAD(H) binding motifs. Ternary complex structures of Rex proteins have been reported in *Thermus thermophilus* (*Tth* Rex, 3IKT), *Thermoanaerobacter ethanolicus* (*Tet* Rex, 3WGI), and *Streptococcus agalactiae* (*Sag* Rex, 3KET) [16,17,18]. Conformational changes upon cofactor binding to *Tth* Rex were proposed to lead to the rotation of the DNA binding domain, resulting in association or dissociation from the Rex binding site [16,19]. In addition, the structural results of *Tet* Rex demonstrated that the new hydrogen bond formation at the last helix upon NADH binding has resulted in the dissociation of the DNA binding domain from the cognate DNA [17]. The Rex proteins have a higher affinity for reduced NADH than oxidized NAD^+^. In high cellular NAD^+^ concentration, Rex-NAD^+^ prefers to bind to the Rex regulon and repress the transcription of target genes [15,20]. When the NADH concentration rises, NADH binding to the *C*-terminal domain of Rex causes a conformational change of the *N*-terminal DNA binding domain, thereby abolishing the formation of Rex and DNA operator sites [16,17].

Our previous studies reported the crystal structures of *Tma* Rex in apo, NAD^+^-bound, and NADH-bound forms. The overall structure of *Tma* Rex was similar to other Rex family proteins and the movement of the *N*-terminal DNA binding domain was based on the last helix, α9 [21]. In the present paper, we determine the crystal structure of *Tma* Rex dimer in complex with an operator DNA and two NAD^+^ molecules. The results show that the winged helix-turn-helix motifs of the *N*-terminal domains bind extensively to DNA and each *C*-terminal dimerization domain contains a NAD^+^ molecule. In addition, a structural comparison of apo, NAD^+^-bound, NADH-bound, and ternary complex forms of *Tma* Rex reveal the life cycle of Rex protein in response to intracellular NAD^+^/NADH ratio.

## 2. Results and Discussion

### 2.1. Model Building and Quality

The ternary complex *Tma* Rex crystals were grown by co-crystallization with NAD^+^ and duplex DNA molecules. The crystal structure of the ternary complex form was determined at 2.40 Å resolution and refined with crystallographic R_work_ and R_free_ values of 19.53% and 25.09%, respectively. The refined model of the ternary complex form included 406 residues of the 2 independent monomers, 2 NAD^+^ molecules, and a 22-bp DNA molecule in the asymmetric unit. The NAD^+^ molecules, and the 22-bp DNA molecule were observed clearly in the 2Fo-Fc maps (Figure S1). The *N*-terminal region (residues 1–4), and *C*-terminal region (residue 208) could not be constructed due to a lack of electron density maps. The refined model of the *Tma* Rex ternary complex showed favored or allowed regions in the Ramachandran plot. The detailed refinement statistics are summarized in Table 1.

### 2.2. Ternary Complex of the T. maritima Rex

**Figure 1:**
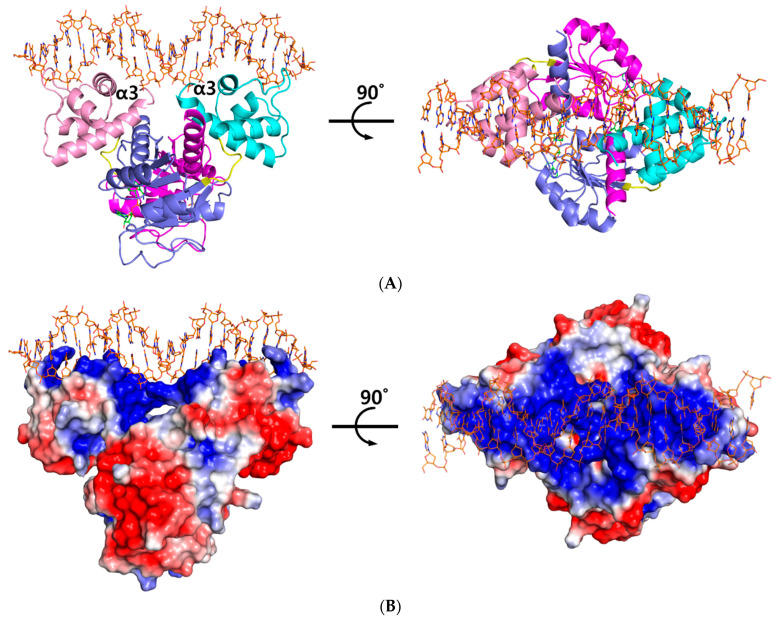
Overall structure of the ternary complex of *Thermotoga maritima* Rex (*Tma* Rex). (**A**) The homo-dimeric *Tma* Rex with NAD^+^ and DNA molecules. The *N*-terminal domains of each subunit are shown in pink and cyan, whereas the *C*-terminal domains are shown in magenta and marine. NAD^+^ and DNA molecules are indicated as green and orange sticks, respectively. DNA recognition helices, α3 helices, are labeled. (**B**) The electrostatic surface diagram of *Tma* Rex. Red represents the negative electrostatic regions and blue represents the positive electrostatic regions. The DNA molecule binds to the top surface of the *N*-terminal domain.

**Figure 2:**
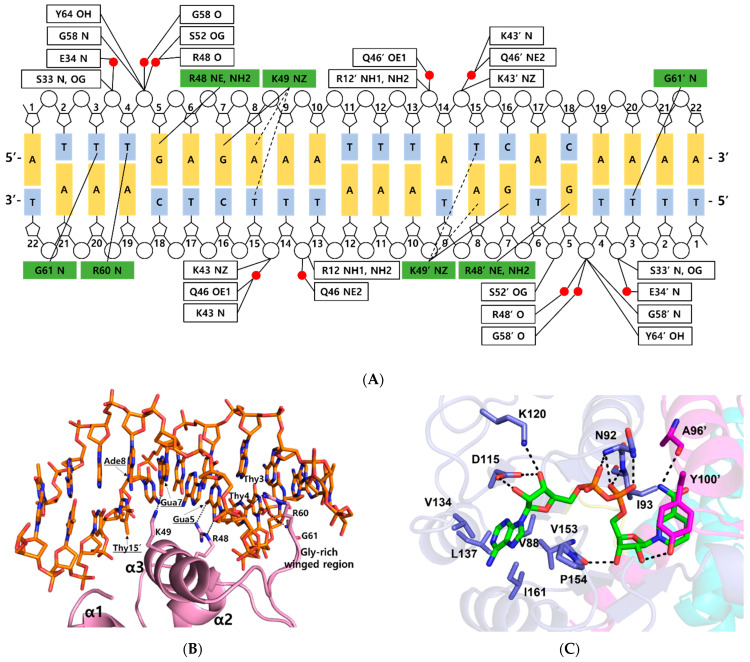
DNA and NAD^+^ binding sites in the ternary complex *Thermotoga maritima* Rex. (**A**) Detailed schematic diagram of DNA interaction in the ternary complex. Residues that interacted with bases are shown in green boxes, and residues that interacted with the phosphate group are shown in white boxes. Water molecules are indicated in red circles. Tight and possible hydrogen bonds are drawn as solid lines and dashed lines, respectively. (**B**) Close-up view of the DNA interaction in the ternary complex. The representation is as described in Figure 1A. Secondary structures involved in DNA interaction are labeled. Residues in major grooves are underlined and indicated with black arrows. Hydrogen bonds are shown as dotted lines. (**C**) NAD^+^ binding mode of the ternary complex. NAD^+^ is shown as a green stick. Residues involved in NAD^+^ binding are labeled and prime indicates the other subunit. Hydrogen bonds are drawn as dotted lines.

The ternary complex structure of *Tma* Rex containing a consensus sequence was solved by molecular replacement at 2.40 Å using a NAD^+^-bound structure (PDB code 5ZZ6) as a model. The *Tma* Rex was composed of two distinct domains, an *N*-terminal DNA binding domain (residues 1–77) and a *C*-terminal dimerization domain (residues 84–208), which was linked by a flexible bending loop (residues 78–83). The asymmetric unit contained a dimeric Rex protein bound to one molecule of NAD^+^ per monomer and a 22-bp DNA of TM0201 operator region (5’-ATTTGAGAAATTTATCACAAAA-3’) (Figure 1A). The *N*-terminal domain of Rex, including the helix-turn-helix motif, formed extensive contacts with the DNA in addition to DNA sequence specificity through electrostatic interactions and hydrogen bonds (Figure 1B). The DNA recognition helix, α3 (residues 43–54), mainly contributed to the interaction with the major groove of DNA by hydrogen bonds. The α1 helix, α2 helix, and a strictly conserved glycine-rich winged region (residues 55–65) also supported various interactions with the DNA molecule. These interactions were almost identical in each subunit. Residues Arg12, Ser33, Glu34, Lys43, Gln46, Arg48, Lys49, Ser52, Gly58, Arg60, Gly61, and Tyr64 interacted with the bases and phosphodiester backbone of DNA through hydrogen bonds and hydrophobic contacts (Figure 2A). These residues are highly conserved in the Rex family, with the exception of Glu34, Ser52, and Arg60.

Within the bacterial order Thermotogales, the Gua5 and Ade8 of the Rex binding site and the binding residues of the Rex family are highly conserved [15]. Residues Arg48 and Lys49 of *Tma* Rex were key residues for recognition of the major groove of the DNA. The NE and NH2 atoms of Arg48 played a role as hydrogen bond donors and interacted tightly with the N7 and O6 atom of Gua5, respectively. The aliphatic side chain of Lys49 helped recognize Ade8 by hydrophobic contacts with the C5 methyl group of the complementary Thy15 (~4.2 Å). The NZ atom of Lys49 interacted tightly with the O6 atom of Gua7 in the DNA. In addition, the NZ atom of Lys49 possibly interacted with the N6 atom of Ade8 and the O4 atom of the complementary Thy15 (~3.7 Å). Therefore, *Tma* Rex recognized the specific DNA operator region using hydrogen bonding and hydrophobic contact with Gua5, Ade8, and nearby bases. Furthermore, residues Arg60 and Gly61 of *Tma* Rex interacted with pyrimidine bases of DNA in the minor groove. The NH1 atom of Arg60 and the N atom of Gly61 were hydrogen bonded with the O_2_ atom of Thy4 and the O_2_ atom of Thy3, respectively (Figure 2B).

Residues Arg12, Ser33, Lys43, Ser52, Gly58, and Tyr64 were involved in ionic or hydrogen bond interactions with the phosphate group of the DNA backbone. In addition, residues Glu34, Lys43, Gln46, Arg48, Ser52, and Gly58 formed water-mediated hydrogen bonds with the phosphate group of the DNA backbone (Figure 2A).

The ternary complex Rex structures were reported from three Rex homologues, *T. thermophilus*, *T. ethanolicus*, and *S. agalactiae*, and they were well matched to the *Tma* Rex with root-mean-square deviation (r.m.s.d.) values of 1.6, 1.3, and 1.7 Å, respectively. Residues Arg48, Lys49, Arg60, and Gly61, involved in the DNA recognition in *Tma* Rex, were highly conserved in Rex homologues. The Rex–DNA interactions were almost identical among ternary complex structures. However, residue Ser45 of *Tma* Rex was not involved in any interaction with DNA while the corresponding residues, Phe43 in *Tth*, Ser47 in *Tet*, and Ala47 in *Sag*, were involved in DNA base interactions [16,17,18].

### 2.3. NAD^+^ Binding Site

The NAD(H) molecule binds to the *C*-terminal domain of *Tma* Rex near the dimer interface. The *C*-terminal domain of *Tma* Rex has the Rossmann fold, which is a nucleotide binding domain, and has a P loop, which is a conserved sequence in Rex homologues. The sequence is Gly-X-Gly-X-X-Gly (residues 89–94; Gly-Ala-Gly-Asn-Ile-Gly).

When the ternary complex of *Tma* Rex was compared with the NAD^+^-bound form (PDB code 5ZZ6), each *C*-terminal domain was well aligned, with an r.m.s.d. of 0.6 Å (Table S1). The NAD binding sites were almost identical and shared the same binding pattern in both structures. The ADP moiety of NAD^+^ was buried in a hydrophobic pocket (residues Val88, Val134, Leu137, Val153, Pro154, and Ile161) and bound by hydrogen bonds with residues Asn92, Ile93, Asp115, and Lys120. The N-ribose moiety of NAD^+^ formed hydrogen bonds with the main-chain atom of Val153, and the hydroxyl group of the Tyr100′ residue, located at the α5 helix of the other subunit. The nicotinamide moiety was bound by a hydrogen bond with the main-chain atom of Ala96′, and formed a π–π interaction with the phenyl ring of Tyr100′ in the other subunit (Figure 2C). The phenyl ring of tyrosine has been experimentally demonstrated to play an important role in the interaction with NAD(H) molecule through site-specific mutagenesis followed by in vitro binding assays [16].

When the *C*-terminal domain of the ternary complex was compared with the NADH-bound form (PDB code 5ZZ7), the domain was structurally aligned with an r.m.s.d. of 1.6 Å (Table S1). In the NADH-bound structure of *Tma* Rex, the ADP moiety of NADH shared the same binding pattern as in the ternary complex structure, whereas the N-ribose and nicotinamide moieties of NADH showed a different binding pattern. The N-ribose moiety of NADH no longer formed any hydrogen bonds with the hydroxyl group of the Tyr100′ residue. Instead, the Tyr100′ residue formed a hydrogen bond with the Asp191 residue of the α9 helix. The reduced nicotinamide moiety of NADH was flipped toward the N-terminus of the α9 helix to make hydrogen bonds with the main-chain atoms of Ile190 and Ile192 residues.

### 2.4. Conformational Changes on DNA Binding

**Figure 3:**
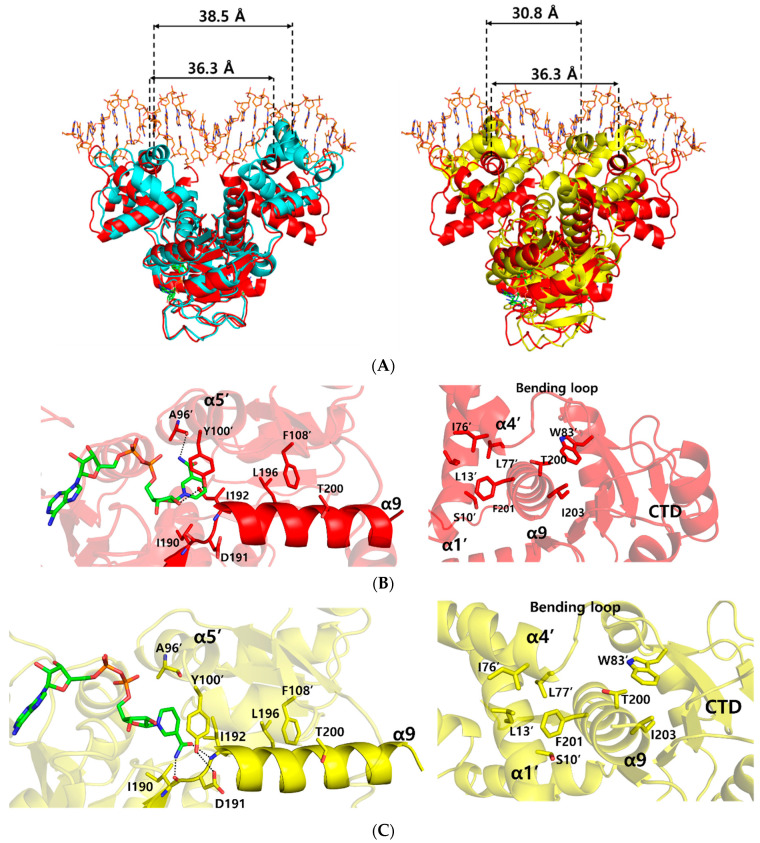
Comparison of the ternary complex structure with NAD^+^-bound and NADH-bound structures of *Thermotoga maritima* Rex (*Tma* Rex). The NAD^+^-bound, NADH-bound, and ternary complex *Tma* Rex forms are shown in cyan, yellow, and red, respectively. (**A**) Superimposition among the structures of NAD^+^-bound, NADH-bound, and ternary complex forms. The distance between DNA recognition helices is based on the Cα atom of the central residue, Arg48, in the α3 helix. (**B**) Representation of the interaction of the α9 helix in the ternary complex. Left panel indicates the NAD^+^ binding sites and the interactions between the *C*-terminal domain and the α9 helix. Right panel indicates the interactions between the *N*-terminal domain and the α9 helix. Hydrogen bonds are shown as dotted lines. (**C**) Representation of the interaction of the α9 helix in the NADH-bound form. Left panel indicates the NADH binding sites and the interactions between the *C*-terminal domain and the α9 helix. Right panel indicates the interactions between the *N*-terminal domain and the α9 helix. Hydrogen bonds are shown as dotted lines.

In order to understand the conformational changes on DNA binding, the structures of the NAD^+^ bound form and ternary complex form were compared by using the DALI server [22]. Although the *N*- and *C*-terminal domains were well aligned structurally with an r.m.s.d. of 1.2, and 0.6 Å, respectively, overall structural comparison gave an r.m.s.d. of 3.7 Å, which represented slight domain movements (Table S1 and Video S1). When compared with each subunit within either the ternary complex or NAD^+^-bound form, each subunit of the ternary complex was almost identical, with an r.m.s.d. of 0.2 Å, whereas each subunit of the NAD^+^-bound form was slightly twisted, with an r.m.s.d. of 3.0 Å (Table S2). These differences indicate that the flexible *N*-terminal domains of NAD^+^-bound *Tma* Rex were stabilized by DNA binding. As a result, the distance between the central residue, Arg48, of DNA recognition helices in the ternary complex structure was 36.3 Å, which was smaller than 38.5 Å in the NAD^+^-bound form (Figure 3A).

To understand the dissociation of *Tma* Rex from a DNA molecule upon NADH binding, we compared the *Tma* Rex structures of the ternary complex and the NADH-bound form. Their *N*- and *C*-terminal domains were fairly well aligned structurally with an r.m.s.d. of 1.9 Å, and 1.8 Å, respectively. However, the overall structural comparison gave a significant difference, with an r.m.s.d. of 4.6 Å, which represented large domain movements (Table S1 and Videos S2 and S3). In *Tma* Rex structures, the α9 helix entered into the other subunit and contributed to dimerization of Rex by forming hydrophobic clusters with the α1 helix, α4 helix, bending loop, and the adjacent *C*-terminal domain (Figure 3B). When NADH molecules were bound in *Tma* Rex, the nicotinamide moiety was flipped and tilted approximately 32° toward the protein hydrophobic core and made hydrogen bonds with the main-chain atoms of Ile190 and Ile192 residues (Figure 3C and Figure S2A). The π–π interaction between the nicotinamide ring and the phenyl ring of Tyr100’ was maintained continuously, which led to translocation of the α5 helix and its following loop containing the Tyr100’ residue (Figure S2B). In addition, the hydroxyl group of Tyr100’ formed a hydrogen bond with the Asp191 residue in the N-terminus of the α9 helix. The Leu196 and Thr200 residues of the α9 helix maintained hydrophobic interactions with the Phe108’ residue. As a result, the α9 helices of both subunits tilted at approximately 10° and the central residue, Phe201, of the α9 helix kept a hydrophobic cluster with the α1 helix, α4 helix, and bending loop at the *N*-terminal domain of the other subunit (Figure 3C and Figure S2C). These conformational changes of the α9 helices caused domain movements of the *N*-terminal domains with a closer distance (30.8 Å) between the central residues of the recognition helix, which is too close to interact with the DNA, resulting in *Tma* Rex dissociating from the DNA (Figure 3A). These conformational changes of Rex upon NADH binding have been seen in several structures of Rex homologues. Detailed information about the conformational changes is summarized in Table S3.

## 3. Materials and Methods

### 3.1. Sample Preparation and Crystallization

DNA cloning, expression, and purification of *Tma* Rex has been recently described by Park et al. [21]. The purified *Tma* Rex was concentrated up to 36 mg mL^−1^ for crystallization using Centricon YM-10 (Millipore). To obtain crystals of the ternary complex *Tma* Rex, 22-bp duplex oligonucleotides (5′–ATTTGAGAAATTTATCACAAAA–3′ and 5′–TTTTGTGATAAATTTCTCAAAT–3′) containing a promoter region of the TM0201 gene regulated by *Tma* Rex were chosen. The oligonucleotides were dissolved in 200 mM NaCl, 1 mM MgCl_2_, 20 mM Tris-HCl at pH 8.0, and 5% (*v/v*) glycerol solution and mixed with *Tma* Rex at a molar ratio of 1.2:2 (1 oligonucleotide: 1 dimeric protein). Crystallization was performed by the sitting-drop vapor diffusion method at 296 K using 96-well CrystalQuick plates (SWISSCI MRC, UK). Each sitting-drop was prepared by mixing equal volumes (0.75 μL) of the reservoir solution and ternary complex. The ternary complex crystals were obtained by co-crystallization with 1 mM NAD^+^ and were grown in 0.05 M Bis-Tris buffer pH 6.5 and 45% (*v/v*) polypropylene glycol P400.

### 3.2. Data Collection

Crystals were transferred to a cryo-protectant solution containing 25% (*w/v*) sucrose and reservoir solution, and then flash-frozen in a stream of liquid nitrogen. X-ray diffraction data of the crystals were collected at 100 K with a DECTRIS Eiger X 16M detector (DECTRIS, Baden, Switzerland) using the synchrotron radiation on beamline BL44XU of the SPring-8 in Japan. Crystals were exposed to X-rays for 1.0 s per image, and 2000 frames were obtained with each 0.1° oscillation. The crystals belonged to the primitive monoclinic space group P2_1_ with unit-cell parameters, a = 69.14 Å, b = 62.84 Å, c = 68.94 Å, *α* = 90.00°, *β* = 108.71°, and *γ* = 90.00°. Data were processed using *XDS* and scaled using the *CCP4* program suite [23,24].

### 3.3. Structure Determination and Refinement

The ternary complex structure of *Tma* Rex containing NAD^+^ and DNA molecules was solved by molecular replacement using the program *PHASER MR* from the *PHENIX* suite using the NAD^+^ bound *Tma* Rex structure (PDB code 5ZZ6) as a search model [21,25]. The structure was built by the *COOT* program and was refined to 2.40 Å resolution with an R_work_ of 19.53% and R_free_ of 25.09% by the *PHENIX* program suite [26,27]. The final model contained a dimeric Rex with two NAD^+^ molecules and a 22-bp DNA molecule in the asymmetric unit. The refined structure was evaluated by *MolProbity* [28]. All data and refinement statistics are summarized in Table 1.

### 3.4. Data Deposition

Coordinate and structure factor of ternary complex *Tma* Rex have been deposited in the Protein Data Bank (PDB; https://www.rcsb.org, 15 December 2021) under accession number 7WB3.

## 4. Conclusions

**Figure 4:**
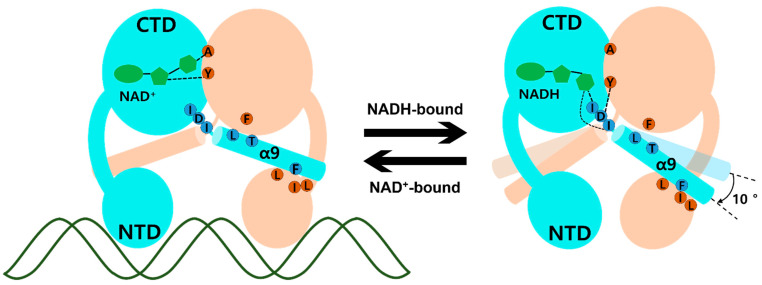
Schematic model of *Thermotoga maritima* Rex (*Tma* Rex) upon binding of NAD(H) molecules. Each subunit of *Tma* Rex is shown in cyan and ivory. The NAD(H) molecules are shown in green in the *C*-terminal domain of one subunit. The duplex DNA molecule is shown as a green line. The highly conserved residues involved in the conformational change are labeled in blue and brown circles. Upon NADH binding, the α9 helices tilted at approximately 10°.

The molecular mechanism of transcriptional regulation in the Rex family has been elucidated. In ternary complex *Tma* Rex, the DNA recognition helix, α3, mainly binds at the major groove of the DNA operator site and the α1 helix, α2 helix, and glycine-rich winged region supported by hydrogen bonding to the duplex DNA. When NADH binds to *Tma* Rex, the nicotinamide ring is flipped and the last helix, α9, is tilted, demonstrating a pendulum-like movement toward the *N*-terminal domain, which results in a dissociation of *Tma* Rex from the DNA operator site. The molecular mechanism of *Tma* Rex also supports the previous models with detailed information, as shown in Figure 4 (Video S4). The pendulum-like rearrangement of the *N*-terminal DNA binding domain shortens the distance between dimer recognition helices, resulting in an unfavorable state toward the DNA operators, a condition that has been commonly found in Rex homologues.

## Figures and Tables

**Table 1:** Data collection and refinement statistics.

Data Set	Ternary Complex of *Tma* Rex
**Data Collection Statistics**	
Space group	P2_1_
Unit-cell parameters	
a, b, c (Å)	69.14, 62.84, 68.94
*α*, *β*, *γ* (°)	90.00, 108.71, 90.00
Wavelength (Å)	0.999994
Resolution (Å)	50.00–2.40 (2.53–2.40) ^a^
Number of observations	84,920
Unique reflections	22,008
Data completeness (%)	99.7 (98.1) ^a^
Redundancy	3.9 (3.9) ^a^
Average I/σ(I)	5.5 (2.1) ^a^
R_merge_ (%) ^ab^	13.9 (44.0) ^a^
**Refinement statistics**	
Resolution (Å)	45.34–2.40
R_work_/R_free_ (%)	19.53/25.09
No. of non-H atoms	4233
Protein	3158
DNA	896
Ligand (NAD^+^)	88
Water	91
Root-mean square deviations	
bonds (Å)	0.002
angles (°)	0.442
Average B-factor (Å^2^)	45.00
Protein	39.24
DNA	66.58
Ligand (NAD^+^)	36.89
Water	40.42
Ramachandran plot (%)	
Favored	98.01
Allowed	1.99
Outliers	0

^a^ Values in parentheses refer to the highest resolution shell. ^b^ R_merge_ = Σ_h_Σ_i_|I(h)_i_−<I(h)>|/Σ_h_Σ_i_ I(h)_i_, where I(h) is the intensity of refection h, Σ_h_ is the sum over all reflections, and Σ_i_ is the sum over i measurements of refection h.

## Data Availability

The data presented in this study are available in the article and Supplementary Materials files.

## Supplementary Materials

The following are available online at https://www.mdpi.com/article/10.3390/ijms23031578/s1.
