# Supplementary material for: Structural Basis of Redox-Sensing Transcriptional Repressor Rex with Cofactor NAD+ and Operator DNA
Source: Int J Mol Sci. 2022 Jan 29;23(3):1578. doi: 10.3390/ijms23031578 (PMC8836258; doi:10.3390/ijms23031578)
Supplement: Supplementary file 1 [file ijms-23-01578-s001.zip › ijms-1534746-Supplementary.pdf]

International Journal of
Molecular Sciences

MDPI

Supplementary Materials

The following are available online at www.mdpi.com/xxx/s1, Figure S1: Electron density maps around NAD⁺ binding site and DNA molecule in the *Thermotoga maritima* Rex, Figure S2: Structural comparison between NADH bound form and ternary complex of *Thermotoga maritima* Rex, Table S1: Structural comparisons among the NAD⁺-bound, NADH-bound, and ternary complex of *Thermotoga maritima* Rex, Table S2: Structural alignments among subunits in NAD⁺-bound, NADH-bound, and ternary complex of *Thermotoga maritima* Rex, Table S3: Structural alignments among subunits in NAD⁺-bound, NADH-bound, and ternary complex of *Thermotoga maritima* Rex, Video S1: Morph conformations between the NAD⁺-bound and ternary complex of *Thermotoga maritima* Rex, Video S2: Morph conformations at the front view between the ternary complex and the NADH-bound form of *Thermotoga maritima* Rex, Video S3: Morph conformations at the top view between the ternary complex and the NADH-bound form of *Thermotoga maritima* Rex, Video S4: Morph conformations about the life cycle of *Thermotoga maritima* Rex in apo, NAD+-bound, ternary complex, and NADH-bound form.

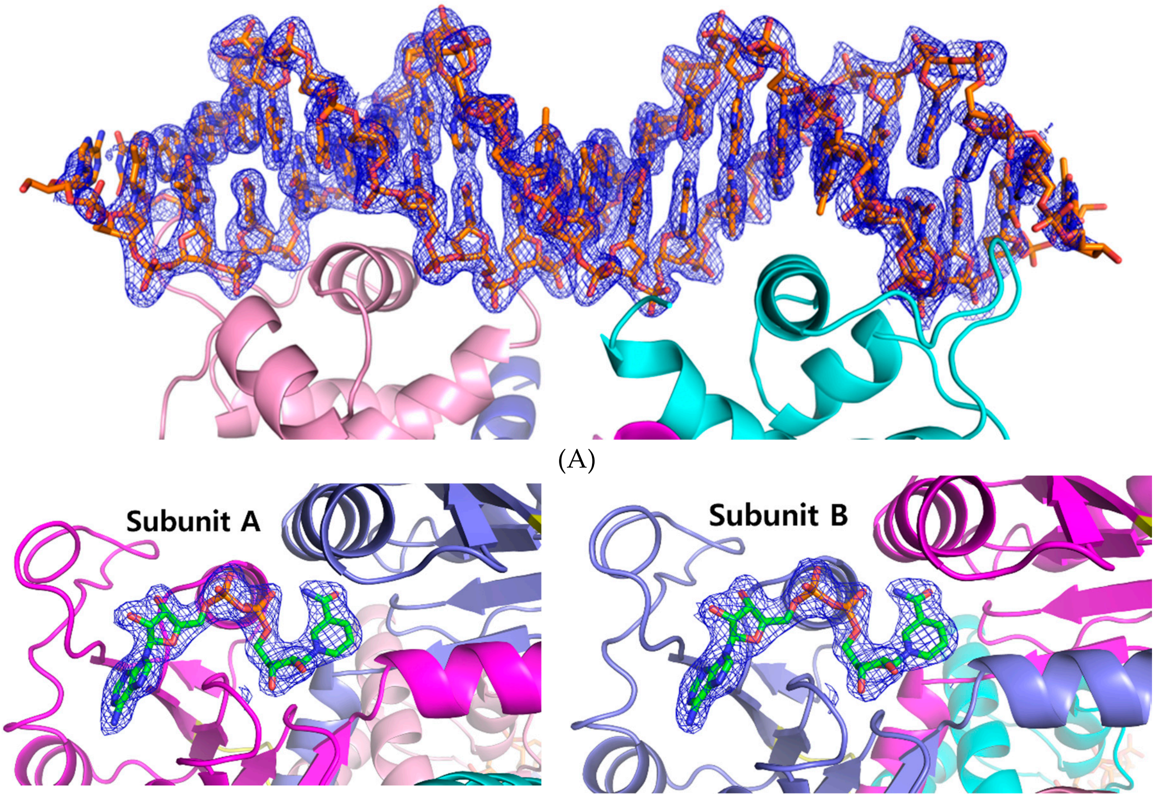

Figure S1. Electron density maps around NAD⁺ binding site and DNA molecule in the *Thermotoga maritima* Rex. 2Fo-Fc maps of the DNA molecule (**A**) and NAD⁺ (**B**), (**C**) are illustrated. Each map is generated at contoured 1.0 σ within 1.6 Å.

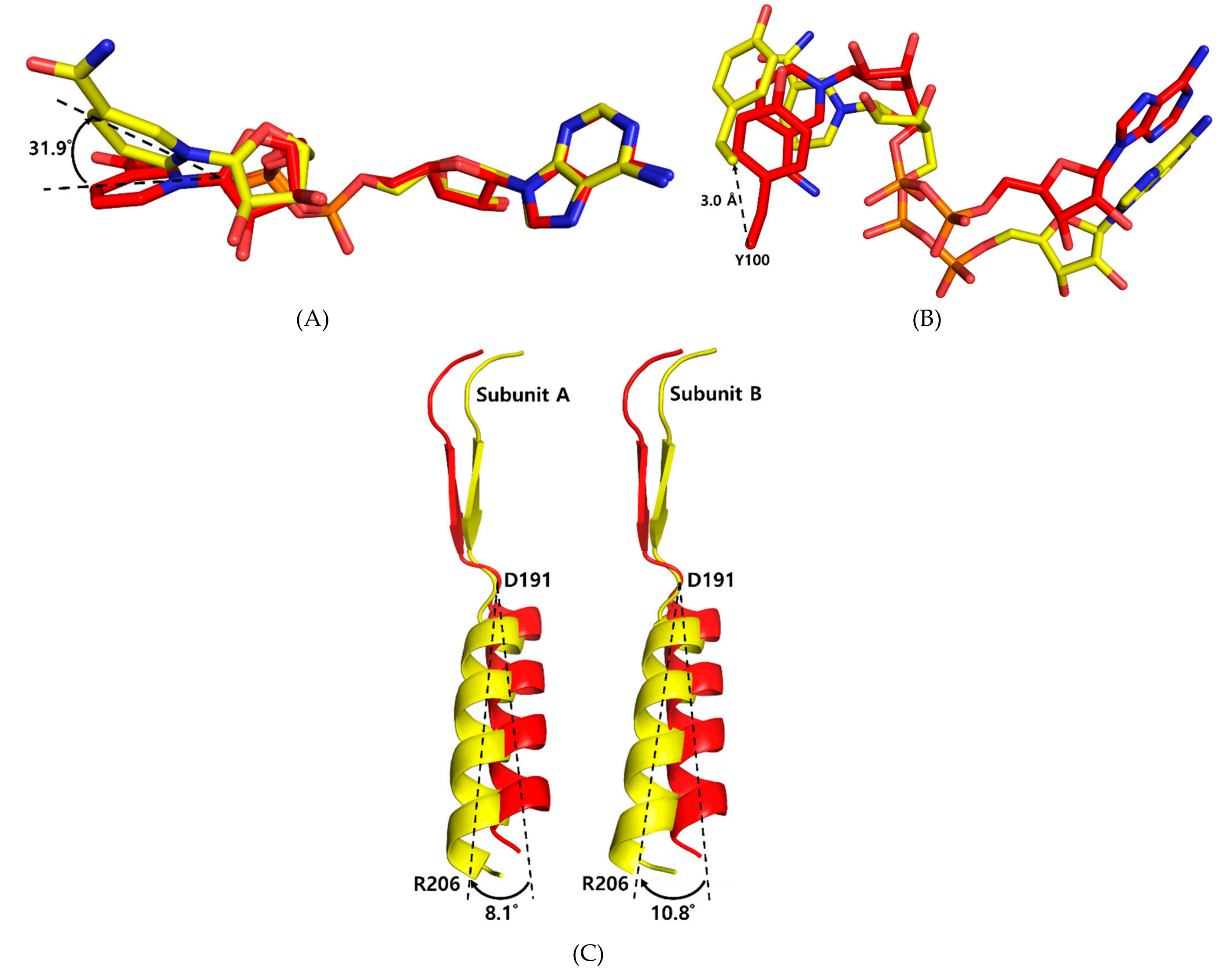

Figure S2. Structural comparison between NADH bound form and ternary complex of *Thermotoga maritima* Rex. Each structure of ternary complex and NADH bound form is shown as red and yellow, respectively. (**A**) The superimposition of NAD(H) molecules. Superimposed cartoon is based on the ADP part of NAD(H). (**B**) Cartoon representation about the translocation of Tyr100. (**C**) The tilted angle of the α9 helix is centered at the residue Asp191 and measured between the residues of Arg206.

Table S1. Structural comparisons among the NAD$^+$-bound, NADH-bound, and ternary complex of *Thermotoga maritima* Rex.

R.m.s.d. (Å)	NAD$^+$-bound	Ternary complex
NAD$^+$-bound	-	
Ternary complex	3.7	-
NADH-bound	5.3	4.6

R.m.s.d. (Å)		NAD$^+$-bound		Ternary complex	
		NTD	CTD	NTD	CTD
Ternary complex	NTD	1.6		-	
	CTD		0.6		-
NADH-bound	NTD	1.2		1.9	
	CTD		1.5		1.8

Table S2. Structural alignments among subunits in NAD^+-bound, NADH-bound, and ternary complex of *Thermotoga maritima* Rex.

R.m.s.d. (Å)	Between Subunit A and B		
	**Full Length**	**NTD**	**CTD**
NAD^+-bound	3.0	1.0	0.5
Ternary complex	0.2	0.1	0.2
NADH-bound	3.1	1.0	0.5

Table S3. Structural alignments among subunits in NAD^+-bound, NADH-bound, and ternary complex of *Thermotoga maritima* Rex. Values refer to the average of measurements between each subunit of Rex homologues.

Data set	*Tma* Rex		*Tth* Rex		*Tet* Rex	
Rotation angle of Nicotinamide ring (°)	32		21.0		16.3	
Cα transition distance of Y100 (Å)	3.0		4.85		3.45	
Cα transition distance of F108 (Å)	3.0		5.25		2.2	
Tilted angle of α9 helix (°)	9.5		15.4		9.05	
Cα distance between R48 of each subunit (Å)	Ternary complex	NADH bound	Ternary complex	NADH bound	Ternary complex	NADH bound
	36.3	30.8	36.9	23.2	36.3	24.2

Video S1. Morph conformations between the NAD^+-bound and ternary complex of *Thermotoga maritima* Rex. The N-terminal domains are shown slight movement upon DNA binding. The animation was created using UCSF Chimera. (https://www.cgl.ucsf.edu/chimera/)

Video S2. Morph conformations at the front view between the ternary complex and the NADH-bound form of *Thermotoga maritima* Rex. The N-terminal domains are shown significant movement upon NADH binding.

Video S3. Morph conformations at the top view between the ternary complex and the NADH-bound form of *Thermotoga maritima* Rex.

Video S4. Morph conformations about the life cycle of *Thermotoga maritima* Rex in apo, NAD^+-bound, ternary complex, and NADH-bound form.
